# Broadly Reactive SARS-CoV-2-Specific T-Cell Response and Participation of Memory B and T Cells in Patients with Omicron COVID-19 Infection

**DOI:** 10.1155/2023/8846953

**Published:** 2023-10-17

**Authors:** Pragya D. Yadav, Rima R. Sahay, Sukeshani Salwe, Diptee Trimbake, Prasad Babar, Gajanan N. Sapkal, Gururaj R. Deshpande, Kiran Bhise, Anita M. Shete, Priya Abraham, Anuradha S. Tripathy

**Affiliations:** ^1^ICMR-National Institute of Virology, Pune, Maharashtra, India; ^2^COVID Hospital, Baner, Pune, Maharashtra, India

## Abstract

January 2022 onward, India witnessed a sudden increase in Omicron COVID-19 infections, having a mild course that prompted us to identify the key host factors/immune molecules modulating disease course/outcomes. The current study evaluated the percentages of lymphocyte subsets by flowcytometry, SARS-CoV-2 specific T-cell immune response by ELISPOT, estimation of plasma cytokine/chemokine levels on a Bio-plex Multiplex Immunoassay System and anti-SARS-CoV-2 IgG levels by enzyme-linked immunosorbent assay in 19 mild Omicron infected patients, 45 mild SARS-CoV-2 (2020) patients and 36 uninfected controls from India. Natural killer cells, B and memory B cells were high in vaccinated and total Omicron-infected patients groups compared to the mild SARS-CoV-2 (2020) patient group, while CD8^+^ T cells were high in total Omicron-infected patients group compared to the uninfected control group (*p* < 0.05 each). Omicron-infected patients had T-cell response against SARS-CoV-2 whole virus, S1 proteins (wild type and delta variant) in 10 out of 17 (59%), 10 out of 17 (59%), and 8 out of 17 (47%), respectively. The current study of Omicron-infected patients elucidates broadly reactive antibody, T-cell response, and participation of memory B and T cells induced by vaccination/natural infection. The limited effect of Omicron's mutations on T-cell response is suggestive of protection from severity. Pro-inflammatory IL-6, IFN-*γ*, chemokines CCL-2, CCL-3, CCL-4, CCL-5, and IL-8 as potential biomarkers of Omicron infection may have future diagnostic importance. The cellular immune response data in Omicron-infected patients with parental Omicron lineage could serve as a starting point to define the readouts of protective immunity against circulating Omicron subvariants.

## 1. Introduction

All viruses change over time, which causes little to no impact on the virus's properties, such as its transmission, associated disease severity, performance of vaccines, therapeutic medicines, diagnostic tools, or other public health and social measures [1]. SARS-CoV-2 has consistently mutated over the course of the pandemic, resulting in variants that are different from the original SARS-CoV-2 virus [2]. The emergence of these variants of interests and variants of concerns (VOCs) during late 2020 posed an increased risk to global public health and the ongoing response to the COVID-19 pandemic. Alpha, Beta, Gamma, and Delta were reported as VOCs causing increased risk to global public health [3]. Since November 26, 2021, when WHO designated the variant B.1.1.529 a VOC as Omicron, researchers around the world have been fiercely carrying out studies to have a better understanding of various aspects of Omicron [3, 4]. Omicron has more than 30 mutations in the Spike protein and 15 in the receptor binding domain (RBD) of the Spike protein that have immune evasive potential [5]. The emergence of Omicron has caused a sharp increase in new COVID-19 infections worldwide, even in vaccinated individuals, raising concerns about immune escape. Omicron was swiftly recognized as being significantly more transmissible than Delta, though it caused less severe disease than Delta, globally [6]. Despite the fact that Omicron poses a lower risk of death and serious illness than the previous SARS-CoV-2 variants, extremely high levels of transmission have resulted in a significant increase in hospitalization, continue to place tremendous strain on health-care systems in the majority of countries, and may cause significant morbidity, especially in vulnerable populations [6, 7].

Epidemiological and other immunological studies could shed light on factors contributing toward the increased number of people testing positive with this variant. Studies on the epidemiology of SARS-CoV-2 Omicron subvariants have shown that the Omicron lineages have advanced mutations in the entire genome, including the spike and RBD regions. All of these heightened mutations have the ability to affect the biological characteristics of the Omicron lineages, causing immunological escape and increased transmissibility compared to the earlier VOCs [8].

There are immunological studies highlighting on the factors, mostly antibodies, contributing toward the increased number of people testing positive with Omicron. Deletions, substitutions, or mutations in the RBD and ACE2 interaction regions may reduce the neutralizing activity of vaccine-induced mAbs, convalescent plasma, and serum, as well as have an effect on antibody binding [9]. The BA.2, BA.2.12.1, and BA.4/5 variants were almost resistant to therapeutic mAbs, casirivimab, idevimab, sotrovimab, cilgavimab, and evusheld in contrast to their substantial neutralizing activity against the previous VOCs [10]. These results collectively indicate that further research into humoral and cellular immune response in Omicron subvariants is necessary. The effectiveness of previous SARS-CoV-2 infections and the effectiveness of vaccines toward reinfection with Omicron are partially explored. The SARS-CoV-2 Omicron variant has continued to evolve with increasing immune escape due to lower neutralizing action by mAbs, vaccination, and past SARS-CoV-2 infection [8, 11].

Cumulatively, these reports suggest that there is a need to explore more about humoral and cellular immune response due to substitutions/deletions/insertions in Omicron subvariants. Thus, a potential threat has been generated due to the emergence of the Omicron variant to public health and economy.

The Omicron variant had been confirmed in 149 countries as of January 6, 2022 [12]. India experienced a sudden increase in COVID-19 cases (120% increase) with the Omicron variant since January 2022, with the severity of the disease being lesser than that observed with other VOCs [12, 13]. Initial reports have highlighted a drastic reduction in the neutralization efficacy of infection and a decrease in the vaccine-elicited antibodies against Omicron. However, is Omicron capable to escape cellular immune responses? And if yes, then to what extent is not yet clear [14]. T-cell responses are a key platform to clear viral infections, to induce B-cell activation for generating antibodies, and to help in providing protection from disease by eradicating virus-infected cells. If mutations in Omicron result in T-cell escape, it could also limit the protection provided by T cells. Six further Omicron subvariants, BQ.1, BA.2.75, CH.1.1, XBB, XBB.1.16, and XBF, are reported as variants under monitoring [15]. Considering the scenario, it is important to identify the key host factors/immune molecules that modulate the disease course and outcome of patients with Omicron COVID-19 infection that seemingly escapes neutralizing antibodies. In order to better understand the dynamics and diversity of cellular immune responses, greater focus should be placed in the future on how mutations affect particular T-cell immune responses. It is important to understand SARS-CoV-2 specific T-cell immunity in the individuals infected with Omicron lineages, as well as in possible future SARS-CoV-2 variants. With this background, the percentages of peripheral lymphocyte subsets (flow cytometry), T-cell response (ELISPOT), and cytokine profile (Bioplex Multiplex platform) were assessed in 19 mild Omicron COVID-19 patients, 45 mild SARS-CoV-2 (2020) patients, and 36 uninfected controls from Pune, Maharashtra, India.

## 2. Materials and Methods

### 2.1. Ethical Approval Declarations

The study was approved by the Institutional Ethical Committee for Research on Humans, based on the guidelines set by the Indian Council of Medical Research, New Delhi. Informed consent had been obtained from all participants.

#### 2.1.1. Study Subjects

The current study was carried out between December 2021 and February 2022. The enrolled Omicron COVID-19 patients (*n* = 19) were mostly the foreign returnees (UAE, South/West/East Africa, Europe, Middle East, USA, and UK) and were confirmed to be positive for Omicron by next-generation sequencing [16, 17]. The participants were categorized into two groups, vaccinated individuals with breakthrough infections (*n* = 15, COVISHIELD (ChAdOx1 nCoV-19), COVAXIN(BBV152), and Pfizer (BNT162b2 mRNA) vaccine) and unvaccinated individuals with 1st time infection (*n* = 4). Detailed characteristics of the Omicron-infected patients are listed in Table 1. The samples from Omicron-infected individuals were collected on 9 (2–17) days post-onset days of symptoms. Thirty-six healthy individuals from the blood donation camps organized by Sassoon General Hospital, Pune, were recruited as uninfected healthy controls for comparison. The uninfected healthy controls were negative for anti-SARS-CoV-2 IgG antibody, as using commercial enzyme-linked immunosorbent assay (ELISA (COVID Kavach-Anti-SARS-CoV-2 IgG Antibody Detection ELISA, M/s Cadila Healthcare Limited, Ahmedabad)) were only included. The healthy controls were unvaccinated.

Out of 36 uninfected subjects, we have immune phenotyping data of 18 uninfected subjects, whereas levels of plasma concentrations of cytokines, chemokines, and growth factors were carried out in all 36. The immune phenotyping data of the Omicron-infected patient group (*n* = 19) were compared with anti-SARS-CoV-2 IgG antibody-negative uninfected controls (*n* = 18) and mild COVID-19 (2020) Indian patients (*n* = 45). Blood samples from the mild COVID-19 (2020) patient group were collected in the acute phase during hospitalization (≤6 days). Anti-SARS-CoV-2 IgG antibody and PRNT levels were determined using S1 RBD, N protein, COVID KAWACH ELISA in the plasma of 19 patients with Omicron infection [13, 14], and the details are provided in *Supplementary 1*.

The immune cell profile and T-cell response were correlated with the IgG antibody levels against anti-SARS-CoV-2 S1-RBD, N protein, and whole virus inactivated antigen.

A schematic representation of the study outline is provided in *Supplementary 2*.

#### 2.1.2. Peripheral Blood Mononuclear Cells (PBMCs) Isolation

Freshly collected 2–3 mL of peripheral blood from the study participants in K3 EDTA tubes were processed for PBMCs isolation by Ficoll-Hypaque (Sigma, USA) density gradient centrifugation method. [18]. It was then resuspended in RPMI-1640 medium (Gibco, Life Technologies, CA USA), supplemented with L-glutamine and sodium bicarbonate. The viability of the cells was >95%, as assessed by staining with 0.1% trypan blue in PBS (Gibco, Life Technologies, CA USA).

### 2.2. Flow Cytometry Analysis

#### 2.2.1. Assessment of Percentages of Natural Killer (NK)/ Natural Killer Like-T (NKT-Like), *B*, T, Memory B, and Memory T Cells

Freshly isolated PBMCs (1 × 10^5^) from Omicron COVID-19 cases (*n* = 19) were used for surface staining of NK cells, NKT-like cells, B cells, T cells, memory B, and memory T cells using cocktail of antihuman antibodies (CD19 PercpCy5.5 (clone HB-19), CD27 PECy7 (clone M-T271), CD3 APCH7 (clone SK7), CD4 BV480 (clone SK3), CD8 FITC (clone RPA-T8), CD45RA PECy7 (clone HI 100), CD62L APC (clone DREG-56), CCR7 PE (clone 2-LI-A) and NK Tritest (CD3 FITC: clone SK7, CD56 PE:clone NCAM 16.2, CD16 PE: clone B73.1, CD 45 RA: C8Mab-1)) all from BD Biosciences following a previously described protocol [18–23]. PE-Cy™7 Mouse IgG1 *κ* Isotype Control (BD Biosciences, San Jose, CA, USA) was used as the negative control.

**Figure 1:**
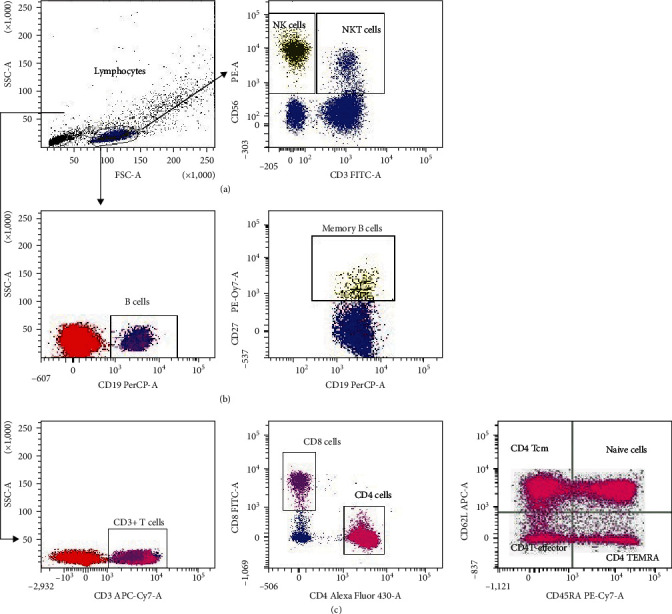
Gating strategy to distinguish different lymphocyte populations by flowcytometry. PBMCs from the study participants were stained with panels of fluorochrome-labeled antibodies to assess the frequency and immune profile. The numbers in the histogram are the mean of the cell population representing for the study group. (a) Lymphocytes and NK cells (CD3^−^CD56^+^), NKT-like (CD3^+^CD56^+^) cells profile; (b) B(CD19^+^) cells and memory B (CD19^+^CD27^+^) cells profile; (c) T-helper (CD3^+^CD4^+^), T-cytotoxic (CD3^+^CD8^+^) cells profile, memory Th and Tc cells, CD4^+^naïve T cells, CD4^+^T central memory cells effector cells, T_EMRA_ cells.

Briefly, PBMCs were incubated with fluorochrome-tagged antihuman monoclonal antibodies for 30 min in the dark. After washing, the cells were fixed with 2% paraformaldehyde. The cells were acquired on FACS Aria II (BD Biosciences, San Jose, CA, USA). For each experiment, 50,000 events were acquired within the lymphocyte gate with appropriate isotype control. Data were analyzed using FACS Diva software (Becton Dickinson, San Jose, CA, USA), and results are expressed as the percentage of positive cells in the gated population. The gating strategy is depicted in Figure 1.

### 2.3. SARS-CoV-2-Specific T-Cell ELISPOT Assay

SARS-CoV-2 specific T-cell response in terms of IFN-*γ* release by ELISPOT assay was performed in Omicron COVID-19 cases (*n* = 19). ELISPOT assay was carried out as previously described [18–20]. To estimate the number of SARS-CoV-2 specific IFN-*γ* secreting spot-forming cells (SFCs), PBMCs were stimulated with gamma-irradiated SARS-CoV-2 whole virus antigen, recombinant S1 protein (wild type) (SARS-CoV-2(2019-nCoV) spike S1(D614G), His Recombinant Protein, Sino Biological, USA) and recombinant S1 protein (delta variant) (SARS-CoV-2 Spike S1 (E154K, L452R, E484Q, D614G, P681R His Recombinant protein Sino Biological, USA)). Wells without any antigen served as negative controls, while those with 10 *µ*g/mL of phytohemagglutinin (Sigma–Aldrich, USA) served as positive controls. All assays were performed in triplicates. The IFN-*γ* SFCs were counted on an ELISPOT reader, customized software (AID GmbH, Strassberg, Germany), and were expressed as the number per 10^5^ cells. The cutoff level for SFCs was set as the average number of SFCs in the negative control wells. Results with high background readings or with no PHA responses were excluded. The number of SFCs in unstimulated wells was subtracted from the number in the antigen-stimulated wells in each subject category for comparison.

### 2.4. Estimation of Cytokine, Chemokine, and Growth Factor Levels

Plasma concentrations of cytokines, chemokines, and growth factors were determined in Omicron-infected patients (*n* = 19) and uninfected controls (*n* = 36) using a Bio-plex Multiplex Immunoassay System (Bio-Rad, Hercules, CA, USA) using a Bio-plex ProTM Human Cytokine 27-plex assay kit as reported previously [24] as per the manufacturer's instructions. Levels of 15 cytokines, including the pro-inflammatory (IL-1*β*, IL-5, IL-6, IL-7, IL-9, IL-15, IL-17, TNF-*α*), anti-inflammatory (IL1-RA, IL-4, IL-10, IL-13), and Th1 (IL-2, IFN-*γ*, IL-12 p70) cytokines along with seven chemokines (Eotaxin, CCL-2, CCL-3, CCL-4, CCL-5, IL-8, CXCL-10) and five growth factors (basic fibroblast growth factor (FGF), G-CSF, GM-CSF, vascular endothelial growth factor (VEGF), platelet-derived growth factor-bb (PDGF-bb)) were estimated. The lowest value of the respective standards was used in the case of undetected concentrations of the cytokines, chemokines, and growth factors in the tested samples [23, 24].

### 2.5. Software and Statistical Analysis

The statistical analyses were performed using IBM SPSS Statistics 25 software (SPSS Inc., Chicago, IL, USA). The Mann–Whitney *U* test was used for the comparison among the study groups. The mean of triplicate experiments in ELISPOT assay was considered for the analysis. Levels of all analytes were analyzed after log_10_ transformation of the observed concentrations of individual cytokines/chemokines/growth factors. Receiver operating characteristic (ROC) analysis was performed using GraphPad Prism 8 software (GraphPad, San Diego, CA, USA). All the data are expressed as median (range). A *p*-value of <0.05 was considered significant. The dot plots were generated on GraphPad Prism 8 software (GraphPad, San Diego, CA, USA).

## 3. Results

### 3.1. Characteristics of the Study Population

The characteristics of the study groups are depicted in Table 1. Most of the study patients were harboring BA.1.1.529 VOC, while three were infected with BA.1, a subvariant of Omicron. The Omicron COVID-19 patients were grouped based on their vaccination status. The study population consists of the following categories: (a) total 19 patients with Omicron infection; (b) 15 vaccinated Omicron-infected patients. These Omicron-infected patients were vaccinated for 119 days for Covishield (ChAdOx1) (*n* = 12), 248 days for COVAXIN (BBV152) (*n* = 1), and 115 days for Pfizer (BNT162 b2 mRNA) (*n* = 2), respectively; (c) four unvaccinated Omicron-infected patients; (d) 45 unvaccinated SARS-CoV-2 (2020) Indian patients with mild infection who eventually recovered and; (e) 36 anti-SARS-CoV-2 IgG antibody negative uninfected healthy controls who were unvaccinated. All the Omicron-infected patients had a milder course of infection and eventually recovered. SARS-CoV-2 (2020) Indian patients with mild infection were used for comparison.

### 3.2. Immunophenotyping in COVID-19 Omicron-Infected Patients

#### 3.2.1. Percentages of NK and NKT-Like Cells

**Figure 2:**
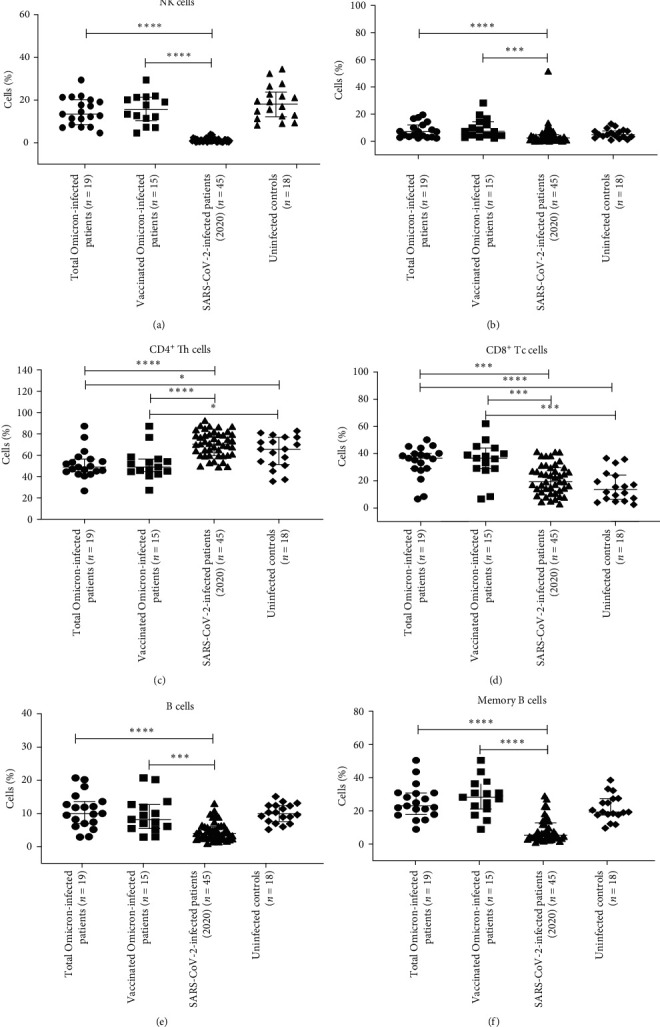
Flow cytometry analysis of NK/NKT-like, B, memory B and IgG+ B cells, T-cell subsets among the study population. PBMCs from (a) total Omicron COVID-19 patients (*n* = 19); (b) vaccinated Omicron-infected patients (*n* = 15); (c) total mild COVID-19 (asymptomatic and mild symptomatic2020) (*n* = 45) patients; (d) uninfected controls (*n* = 18) were stained and acquired on a flow cytometer. Vertical scatter plots denote the comparisons of frequencies of immune cells and their subpopulation among different study groups: (a) NK cells; (b) NKT-like cells; (c) B cells; (d) memory B cells; (e) CD4^+^T-helper cells; (f) CD8^+^T-cytotoxic cells profile. Data are presented as percentage of immune cells out of lymphocytes. The dots represent individual values and bars represent mean + SD values ( ^*∗*^*p*-value < 0.05,  ^*∗*^ ^*∗*^*p*-value < 0.005, and  ^*∗*^ ^*∗*^ ^*∗*^*p*-value < 0.0001).

The percentages of NK and NKT-like cells were significantly higher in the total Omicron COVID-19 patients group and vaccinated Omicron COVID-19 patients group compared to total mild SARS-CoV-2 patients (2020) group (*p* < 0.05 in each) (Table 2, Figure 2). However, the percentages of NK and NKT-like cells were comparable among total Omicron COVID-19 patients and uninfected control groups (Table 2, Figure 2).

#### 3.2.2. Percentages of B and Memory B Cells

The percentages of B and memory B cells were significantly high in both in total Omicron COVID-19 patients group and vaccinated Omicron COVID-19 patients group compared to the total mild SARS-CoV-2 patients (2020) group Table 2, Figure 2) (*p* < 0.05 in each).

#### 3.2.3. Percentages of CD4^+^, CD8^+^ T Cells and Memory T-Cell Subsets

The percentage of CD4^+^Th cells was significantly lower, while CD8^+^ Tc cells were significantly higher in the total Omicron COVID-19 patients group and the vaccinated Omicron COVID-19 patients group compared to the total mild SARS-CoV-2 patients (2020) group and uninfected control (Table 2, Figure 2) (*p* < 0.05 in each).

Further, the homeostatic distribution of CD4^+^ and CD8 ^+^T cells based on CD62L and CD45RA expression in terms of naïve (CD62L^+^CD45RA^+^)/memory (CD62L^+^CD45RA^−^)/effector memory (CD62L^−^CD45RA^−^)/terminally differentiated (CD62L^−^CD45RA^+^) subsets were analyzed among the study groups.

**Figure 3:**
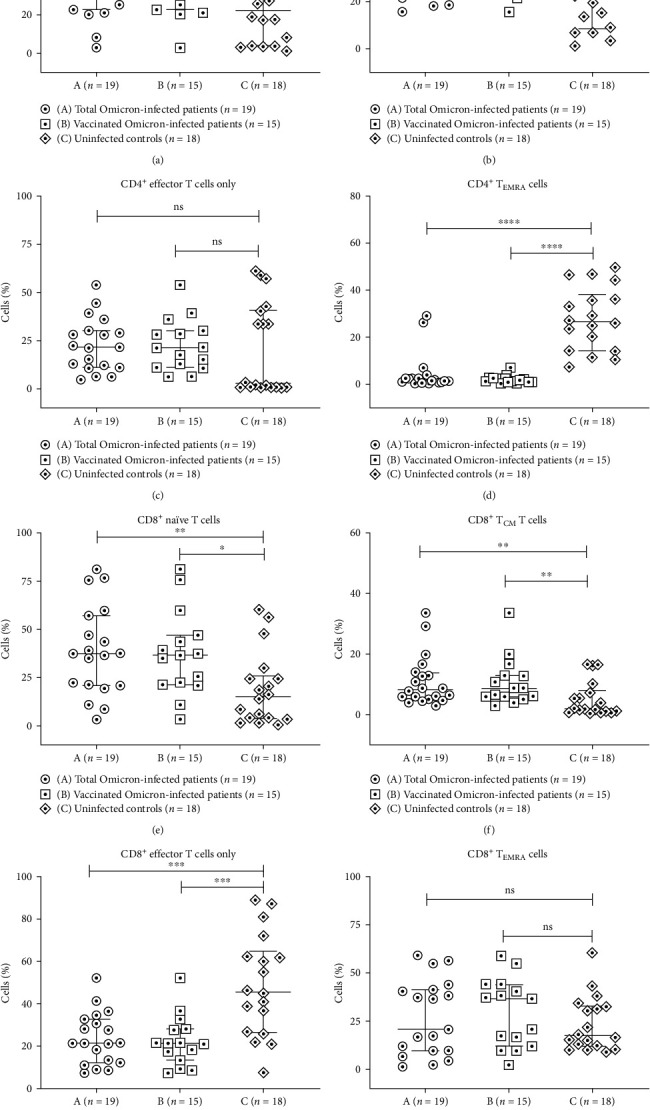
Flow cytometry analysis of T and memory T-cell subsets in Omicron-infected patients and uninfected controls. PBMCs from (A) total Omicron-infected patients (*n* = 19); (B) vaccinated Omicron-infected patients (*n* = 15); (C) uninfected controls (*n* = 18) were stained and acquired on flow cytometer. Vertical scatter plots denote the comparisons of frequencies of immune cells and their subpopulation among different study groups: (a–d) CD4^+^ memory T-cell subsets (e–h) CD8^+^ memory T-cell subsets, namely naive, central, T_EMRA_ and effector memory. Data are presented as percentage of immune cells out of lymphocytes. The dots represent individual values and bars represent mean + SD values ( ^*∗*^*p*-value < 0.05,  ^*∗*^ ^*∗*^*p*-value < 0.005, and  ^*∗*^ ^*∗*^ ^*∗*^*p*-value < 0.0001).

In CD4^+^ Th-cell compartment, the percentage of the central memory population was found to be significantly high in the vaccinated Omicron COVID-19 patient group compared to the uninfected controls (*p* < 0.05) (Table 2, Figure 3). The percentages of CD4^+^T_EMRA_ cells were significantly low in the total Omicron-infected patient group and in the vaccinated Omicron COVID-19 patient group compared to the uninfected controls (*p* < 0.05 in each) (Table 2, Figure 3). However, the percentages of naïve and effector CD4^+^ memory T cells were comparable among unvaccinated total and vaccinated Omicron COVID-19 patient groups and uninfected controls (Table 2, Figure 3).

In the CD8^+^ T-cell compartment, the percentages of naïve and central memory populations were significantly higher, while the effector memory population was significantly lower in vaccinated and total Omicron COVID-19 patient groups compared to the uninfected control group (*p* < 0.05 in each) (Table 2, Figure 3). The percentages of CD8^+^T_EMRA_ cells were comparable between Omicron infected (both vaccinated and total) and uninfected control groups (Table 2, Figure 3).

#### 3.2.4. Correlation between Anti-SARS-CoV-2 IgG Antibody Levels and CD8^+^ Terminally Differentiated T and Memory B Cells Frequencies

A lack of correlation was observed between anti-SARS-CoV-2 IgG antibody levels against the whole virus-inactivated antigen and the percentages of CD4^+^ naïve cells (*R* = −0.4995, *p*=0.0294). A positive correlation was observed between anti-SARS-CoV-2 IgG antibody levels against the whole virus-inactivated antigen and the percentages of CD8^+^ terminally differentiated T cells (*R* = 0.5034, *p*=0.028) and memory B cells (*R* = 0.6127, *p*=0.0053) (Table 3, *Supplementary 1*).

### 3.3. SARS-CoV-2-Specific T-Cell Response

**Figure 4:**
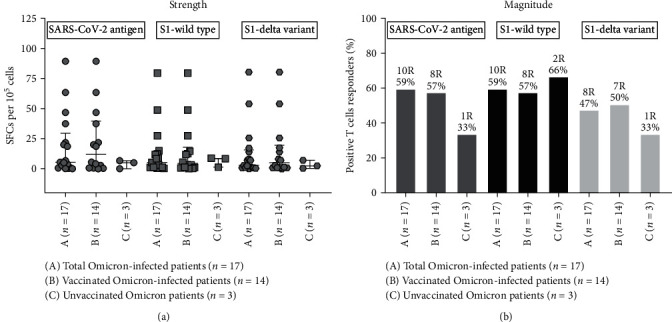
Strength and magnitude of SARS-CoV-2-S1 specific IFN-*γ* producing T-cell response among Omicron-infected patients. PBMCs isolated from all subjects were cultured with gamma-irradiated SARS-CoV-2 whole virus antigen, recombinant S1 protein (wild type), and recombinant S1 protein (delta variant) in vitro. IFN-*γ* secreting cell frequencies were determined by ELISPOT assay. (a) Strength in terms of SFC per 10^5^ cells and (b) magnitude of the SARS-CoV-2-S1 specific IFN-*γ* producing T-cell response in terms of percentage. (A) Total Omicron-infected patients (*n* = 17); (B) vaccinated Omicron-infected patients (*n* = 14); (C) uninfected Omicron patients (*n* = 3).

To determine the SARS-CoV-2 specific T-cell response in terms of IFN-*γ* release, we performed ELISPOT assay using gamma irradiated SARS-CoV-2 whole virus antigen, recombinant S1 protein (wild type) and recombinant S1 protein (delta variant) as recall antigens. In the Omicron COVID-19 patient group (*n* = 17) (14 vaccinated, 3 unvaccinated), IFN-*γ* responses in unstimulated, gamma irradiated SARS-CoV-2 whole virus antigen stimulated, SARS-CoV-2 S1-(wild-type) stimulated, recombinant S1 protein (delta variant) stimulated and PHA-stimulated cells of (a) 12 COVISHIELD vaccinated Omicron-infected patients were 2.14 (0–17.6), 21.6 (0–89.3), 12.3 (0.33–79.3), 11.8 (0–80.3), and 167 (36.3–378.3) SFCs/10^5^cells; (b) single COVAXIN-vaccinated Omicron-infected patient was 9.67, 46.67, 48.67, 54.00, and 248.7 SFCs/10^5^cells; (c) single Pfizer-vaccinated Omicron-infected patient was 0.67, 3.67, 12.33, 17.33, and 242.3 SFCs/10^5^cells; (d) three unvaccinated Omicron-infected patient were 0.75 (0.17–1.33), 4.1(0–6.67), 4.9 (1.33–8.67) 3 (0.33–8.67) and 141 (21.3–141.7) SFCs/10^5^cells, respectively (Figure 4). Notably, a single Pfizer-vaccinated Omicron-infected patient and one of the four unvaccinated Omicron-infected patients had spontaneous IFN-*γ* responses in unstimulated as 40.6 and 24 SFCs/10^5^ cells, respectively. Hence, the data of these two were excluded from analysis, and the data of only 17 Omicron COVID-19 patients were taken into account.

#### 3.3.1. Strength of the SARS-CoV-2-Specific T-Cell Response

SFCs/10^5^cells against gamma irradiated SARS-CoV-2 whole virus antigen, SARS-CoV-2 S1-(wild type), recombinant S1 protein (delta variant) in (a) 12 vaccinated Omicron-infected patient group were 19.5 (0–89.3), 10.2 (0–70.3), and 9.69 (0–80.3) SFCs/10^5^cells; (b) single COVAXIN-vaccinated Omicron-infected patient was 37, 39, and 44.3 SFCs/10^5^cells; (c) single Pfizer-vaccinated Omicron-infected patient was 3,11.6 and 16.6 SFCs/10^5^cells; (d) three unvaccinated Omicron-infected patient were 3.17 (0–6.67), 8.33, and 3.5 (0.33–6.67) SFCs/10^5^cells, respectively (Figure 4(a)).

#### 3.3.2. Magnitude of the SARS-CoV-2-Specific T-Cell Response

Ten out of 17 (59%), 10 out of 17 (59%), and 8 out of 17 (47%) Omicron-infected patients displayed T-cell response against SARS-CoV-2 whole virus antigen, S1 protein (wild type) and S1 protein (delta variant), respectively (Figure 4). It is important to note that 8 out of 14 (57%), 8 out of 14 (57%), and 7 out of 14 (50%) vaccinated Omicron-infected patients displayed functional SARS-CoV-2 specific T-cell response against SARS-CoV-2 whole virus antigen, S1 protein (wild type) and S1 protein (delta variant), respectively. Three unvaccinated Omicron-infected patients displayed functional SARS-CoV-2 specific T-cell response to at least one recall antigen. This scenario of SARS-CoV-2 specific T-cell response indicated the presence of vaccine /infection-induced broad T-cell immunity in Omicron infection (Figure 4(b)).

### 3.4. Peripheral Cytokines and Chemokines in Omicron-Infected Patients and Uninfected Controls

**Figure 5:**
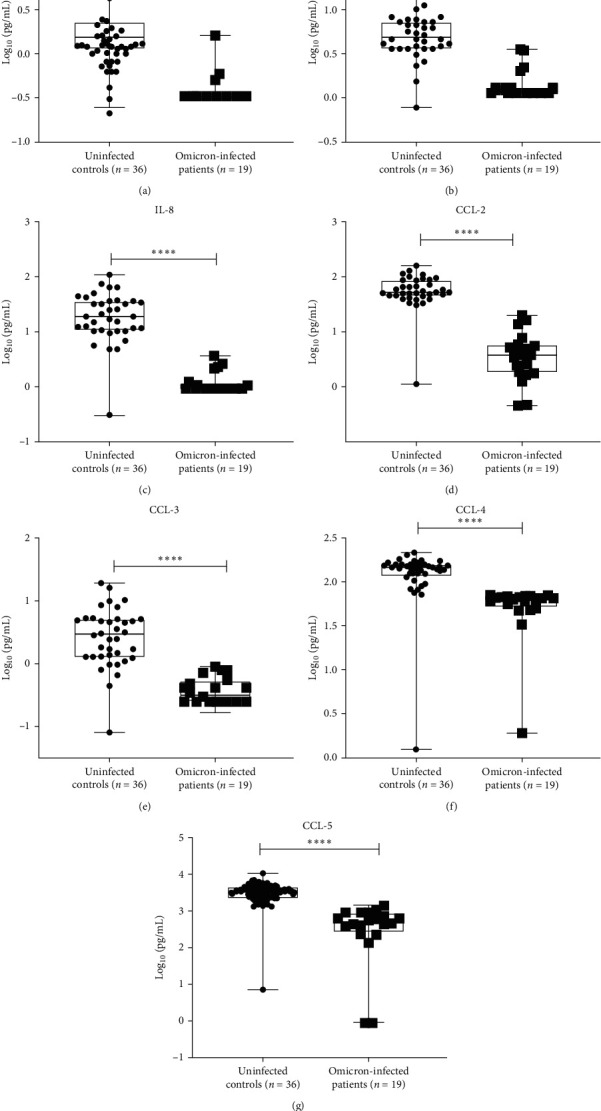
Concentrations of cytokines and chemokines expressed in log_10_ (pg/mL) in Omicron-infected patients and uninfected controls. Comparison of concentrations of cytokines and chemokines expressed in log_10_ (pg/mL) in Omicron-infected patients and uninfected controls. (a) IL-6, (b) IFN-*γ*, (c) IL-8, (d) CCL-2, (e) CCL-3, (f), CCL-4, (g) CCL-5;  ^*∗*^*p*-value < 0.05,  ^*∗*^ ^*∗*^*p*-value < 0.005, and  ^*∗*^ ^*∗*^ ^*∗*^*p*-value < 0.0001).

Pro-inflammatory cytokines IL-6, IFN-*γ* and chemokines CCL-2, CCL-3, CCL-4, CCL-5, and IL-8 were significantly elevated in Omicron-infected patients compared to uninfected healthy controls (*p* < 0.0001 in each) (Table 4, Figure 5). The levels of other cytokines, chemokines, and growth factors namely IL-5, IL-7, IL-9, IL-15, IL-17, TNF-*α*, IL-10, IL-2, Eotaxin, CXCL-10, FGF basic, GM-CSF, and PDGF-bb were significantly lower in Omicron infected patients compared to uninfected healthy controls (*p* < 0.0001 in each) (Table 4, Figure 5).

#### 3.4.1. ROC Analysis of Cytokines

**Figure 6:**
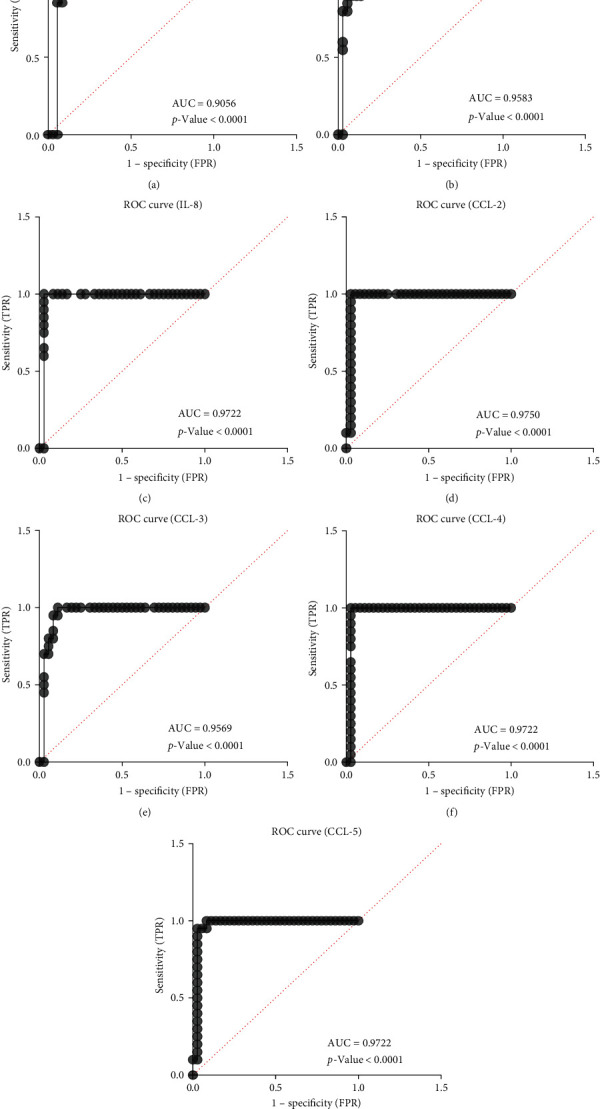
Receiver operating characteristic (ROC) for IL-6, IFN-*γ*, IL-8, CCL-2, CCL-3, CCL-4, CCL-5, and IL-8. ROC curves for IL-1*β*, TNF-*α*, CXCL-10, and IL-4, validating their applicability as biomarkers of recent infection. The ROC characteristics of (a) IL-6: cutoff: 0.33 pg/mL, sensitivity: 95%, specificity: 91.67%, AUC = 0.9056; (b) IFN-*γ*: cutoff: 3.48 pg/mL, sensitivity: 100%, specificity: 86.11%, 0.9583; (c) IL-8: cutoff: 2.61 pg/mL, sensitivity, 100%: specificity: 97.22%, AUC = 0.9722; (d) CCL-2: cutoff: 13.78 pg/mL, sensitivity: 100%, specificity: 97.22%, AUC = 0.9750, (e) CCL-3: cutoff: 0.25 pg/mL, sensitivity: 100%, specificity: 88.89%, AUC = 0.9569; (f) CCL-4: cutoff: 69.53 pg/mL, sensitivity: 100%, specificity: 97.22%, AUC = 0.9722; (g) CCL-5: cutoff: 4547.35 pg/mL, sensitivity: 100%, specificity: 91.67%, AUC = 0.9722. The area under the curve (AUC) values of the above analytes were greater than 0.9 (*p* < 0.0001), which is indicative of higher diagnostic value. The high sensitivity and specificity of the analytes' cutoffs suggest that they can potentially act as biomarkers of Omicron infection.

To generate a cutoff for the analytes among the patients and uninfected healthy controls, ROC analysis was performed (Table 5, Figure 6). The ROC analysis generated cutoffs for individual cytokines/chemokines that could be used to distinguish uninfected healthy controls from patients with Omicron infection and might have future diagnostic importance.

The ROC characteristics of IL-6 and IFN-*γ* included cutoff values of 0.33 and 3.48 pg/mL, respectively (sensitivity: 95% and 100%, respectively; specificity: 91.67% and 86.11%, respectively). Similarly, the ROC characteristics of chemokines namely CCL-2, CCL-3, CCL-4, CCL-5, and IL-8 showed cutoff values of 13.78, 0.25, 69.53, 4,547.35, and 2.61 pg/mL, respectively (sensitivity, 100% for each chemokine; specificity, 97.22%, 88.89%, 97.22%, 91.67%, and 97.22% respectively). The area under the curve (AUC) values of the above analytes were greater than 0.9 (*p* < 0.0001), which is indicative of higher diagnostic value. Thus, the pro-inflammatory cytokines IL-6 and IFN-*γ* and the chemokines CCL-2, CCL-3, CCL-4, CCL-5, and IL-8 were found to be predictive of Omicron infection, which was confirmed by ROC analysis (AUC = 0.9056, 0.9583, 0.9750, 0.9569, 0.9722, 0.9722, and 0.9722, respectively). The high sensitivity and specificity of the analytes' cutoffs suggest that they can potentially act as biomarkers of Omicron infection (Tables 4 and 5, Figures 5 and 6).

## 4. Discussion

This investigation is one of the few to examine clinical and immunological profiles of individuals infected with Omicron SARS-CoV-2 from India, a major site for COVID-19 infection. All the enrolled symptomatic patients, whose samples were collected between the time frame 2–17 PODs, were experiencing similar symptoms at the time of sampling, and hence their immune responses were expected to be consistent. Liu et al. [25] have reported neutralizing antibody activities from two doses of ChAdOx1 nCoV-19, mRNA-1273, or MVC-COV1901 immunizations followed by a booster dose of mRNA-1273, which were able to induce detectable neutralizing antibodies against the Omicron variant. They have suggested that in addition to neutralizing titers, T-cell response may play a role in vaccine effectiveness [25]. In another study by Dimeglio et al. [26], where the authors compared the concentrations of binding antibodies before breakthrough infections with Delta or Omicron SARS-CoV-2 variants and suggested that infections with the Omicron variant can occur despite high binding antibody concentrations.

Our T-cell data are in line with the aforementioned recommendations and with the related report by Keeton et al. [27], suggesting that Omicron's mutations have a limited impact on the T-cell response irrespective of vaccination or prior infection status and may still offer significant protection from severe disease. Camilla Mattiuzzi et al. [28] reported that lower severity of illness caused by the SARS-CoV-2 Omicron variant and the efficacy of vaccination contributed to lower SARS-CoV-2 pathogenicity. The detected reduction in the percentages of CD4^+^ Th cells and rise in the percentages of CD8^+^ Tc cells in the total Omicron COVID-19 patients' group and in the vaccinated Omicron COVID-19 patients group could be suggestive of a skewed immune response toward a more cytotoxic cellular immune response. This could be representing a scenario of the immune system's overall balance due to the disease/vaccination status. In a similar way, the detection of higher CD8^+^ T cells, CD8^+^ naïve T-cell subsets along with broadly reactive vaccine-generated T-cell response in vaccinated individuals of the Omicron-infected group could be associated with a milder course of infection. Observed higher B and B memory cells, crucial to prolonged protection after vaccination, and higher NK and NKT-like cells responsible for a robust adaptive immune response could be the molecules for disease resolution. Further key finding of our data is that both vaccinated and total Omicron-infected patients displayed similar T, B, and NK cells immune responses following infection. Overall, this cumulative scenario has led to the generation of an Omicron-induced immune response that has resulted in the manifestation of a mild course of infection irrespective of the vaccination status of the patients.

Antibodies and memory B cells are considered correlates of protection against various viral infections [29]. However, anti-SARS-CoV-2 IgG antibodies may not be serving the purpose as evident from the reports of reinfection in seropositive recovered/ breakthrough infections [30, 31]. A lack of correlation between anti-IgG antibody levels against an antigen and the percentages of CD4^+^ naive T cells could be related to different types of immune responses generated and also to the factors responsible for the complexity of the immune system. B cells are primarily accountable for producing anti-IgG antibodies against a particular antigen. CD4^+^ naive T cells are a subset of T cells and are vital for directing and controlling the immune response to different infections. These discrete immune system components can react to different stimuli independently. SARS-CoV-2 could trigger B cells to produce anti-IgG antibodies. On the other hand, memory B cells may be in charge of generating anti-IgG antibodies upon re-exposure if the person has already been exposed to the antigen. Nevertheless, it is possible that the secondary immune response does not involve CD4^+^ naive T cells as much. This could result in no association between the two parameters. Thus, the diversity in immune mechanisms, immunological memory, and antigen specificity could be the factors responsible for the lack of correlation between SARS-CoV-2 specific anti-IgG antibody levels and the percentages of CD4^+^ naive T cells in this study.

CD4^+^ T_EMRA_ cells are a subset of memory T cells that have previously encountered a pathogen and are ready to respond more quickly upon reinfection. However, their precise role in various viral infections can vary. The behavior of CD4^+^ T_EMRA_ cells can be complex and context-dependent. They can play both beneficial and detrimental roles in different viral infections. Anft et al. [32] have reported lower frequencies of terminally differentiated T-cell subsets in patients with both severe and critical COVID-19 disease. Further, a report by Jae Jung et al. [33] showed lower expression of T_EMRA_ cells in COVID-19 convalescent patients for 10 months, indicating that it may take some time for the immune system to normalize postinfection with COVID-19. Thus, the current data of lower phenotypic CD4^+^ T_EMRA_ cells during Omicron COVID-19 infection is an expected phenomenon. A positive correlation of IgG antibody levels against the whole virus-inactivated antigen and the percentages of CD8^+^ terminally differentiated T and memory B cells (Table 3) in the currently studied patients suggest the need to look for multiple correlates of protective immunity against COVID-19.

Our ELISPOT data displayed functional SARS-CoV-2 specific T-cell response irrespective of the recall antigen, indicating the presence of vaccine-induced broad T-cell immunity in vaccinated and total Omicron-infected patients. It could have implications for understanding the robustness of immunity following natural infection and/or vaccination. The emergence of pro-inflammatory cytokines IL-6 and IFN-*γ* and chemokines CCL-2, CCL-3, CCL-4, CCL-5, and IL-8 as potential biomarkers of Omicron infection might have future diagnostic importance [24] Wong et al. [34] reported in a Chinese population that IFN-*γ*, IL-12, IL-1*β*, and IL-6 can induce hyper-innate inflammatory response due to invasion of the respiratory tract by SARS-CoV, leading to the activation of Th1-cell-mediated immunity by the stimulation of NK and cytotoxic T lymphocytes. In a similar line, our study demonstrated elevated levels of IL-6 and IFN-*γ*, which might induce hyper-innate inflammatory response and might be leading to a higher percentage of NK cells in both vaccinated and total Omicron-infected individuals. It is important to note that CCL5/RANTES is a chemokine important for T-cell homing and migration during acute virus infections. [35, 36]. Elevated levels of CCL-5 in our study might have a similar role in the group of Omicron-infected patients.

The absence of antigen-specific immune cell profile data and immune response data of vaccinated mild SARS-CoV-2 (2020) patients could be taken as the limitations of our study. Despite that, our study demonstrated some key findings on immune response in patients with Omicron infection.

## 5. Conclusion

In summary, our study in Omicron-infected SARS-CoV-2 patients elucidates broadly reactive antibodies as well as T-cell response and participation of memory B and T cells induced by vaccination or natural infection, which could be contributing toward protection from severe COVID-19. The current data of cellular immune response in Omicron-infected patients with parental Omicron lineage may form the basis to define the readouts of protective immunity against circulating Omicron subvariants.

## Figures and Tables

**Table 1:** Characteristics of the study population.

Parameters	Vaccinated Omicron-infected patients			Unvaccinated Omicron-infected patients	Unvaccinated SARS-CoV-2 (2020) Indian patients with mild infection	Uninfected controls
Study population	*n* = 15			*n* = 4	*n* = 45	*n* = 36

Age (median, range)	42 (18–72)			17 (7–28)	38 (15–75)	20 (18–26)

Gender (male/female)	8M/7F			0M/4F	24M/21F	26M/10F

Vaccination status	Covishield (ChAdOx1 nCoV-19)	COVAXIN (BBV152)	Pfizer (BNT162b2 mRNA)	Unvaccinated	Unvaccinated	Unvaccinated
	*n* = 12 (6M/6F)	*n* = 1 (M)	*n* = 2 (1M/1F)			

Status of infection	Asymptomatic (*n* = 9) Mild symptomatic (*n* = 3)	Asymptomatic (*n* = 1)	Asymptomatic (*n* = 1) Mild symptomatic (*n* = 1)	Asymptomatic (*n* = 3) Mild symptomatic (*n* = 1)	Asymptomatic (*n* = 23) Mild symptomatic (*n* = 22)	NA ^*∗*^
Days post onset of illness (POD)	9 (2–17) days	NA	3 days	12 days	7 (0–17)	
Status of reinfection	*n* = 2	*n* = 1	No	No	No	
Infection post-vaccination days	119 (16–222)	248	115 (137–219)	NA ^*∗*^	NA ^*∗*^	
Comorbid conditions (diabetes/HTN)	*n* = 3	No	No	No	No	

S1RBD IgG status and titer	Positives (*n* = 12,100%) titer (1,000–>3,200)	Positive (*n* = 1) titer (2,000)	Positives (n = 2) titer (800–>3,200)	Positive (*n* = 2) titer (100–2,000)	NA	Anti-SARS-CoV-2 IgG antibody negatives
N Protein IgG status and titer	Positives (*n* = 10, 83.3%) titer (50–>3,200)	Positive (*n* = 1) titer (3,200)	Positive (*n* = 1) titer (50)	Negatives (*n* = 4) titer (<50)		
COVID KAWACH IgG status and titer	Positives (*n* = 10, 83.3%) titer (100–>3,200)	Positive (*n* = 1) titer (3,200)	Positive (*n* = 1) titer (100)	Positives (*n* = 3) titer (100–6,400)	Positives (*n* = 45) titer (NA)	

All the Omicron-infected cases had a milder course of infection and recovered eventually. M, male; F, female; NA, not-available; NA ^*∗*^, not applicable; HTN, hypertension/hypotension (blood pressure).

**Table 2:** Immune cells profile in Omicron COVID-19 patients.

	Total Omicron-infected patients (*n* = 19)	*p* Value^a^	Vaccinated Omicron-infected patients (*n* = 15)	*p* Value^b^	Total mild SARS-CoV-2 patients (asymptomatic and symptomatic 2020)	*p* Value^c^	Uninfected controls (*n* = 18)	*p* Value^d^	*p* Value^e^
NK and NKT-like cell profile									
NK cells	13.4 (8.5–20.1)	ns	17.7 (11.4–21.5)	ns	1.3 (0.6–2.8)	0	18.1 (12.2–23.73)	0.0001	0.0001
NKT-like cells	7.1 (3.1–12)	ns	7.4 (4.2–14.4)	ns	3.1 (0.8–7.6)	0.016	4.9 (3.15–7.7)	0.0007	0.0003
B-cell profile									
B cells	10 (7–13.6)	ns	8.2 (5.5–12.7)	ns	4.5 (2.9–6.4)	0	9.95 (7.525–12.4)	0.0001	0.0002
Memory B cells	23 (17.8–30.8)	ns	28.2 (21.1–36.3)	ns	6.9 (3.6–17.2)	0	19.35 (17.58–27.35)	0.0001	0.0001
T-cell Profile									
CD4^+^Th cells	48.9 (44.5–56.4)	0.0176	48.9 (44.5–56.4)	0.0372	69.5 (59.7–78.3)	0	65.65 (51.23–76.85)	0.0001	0.0001
CD8^+^Tc cells	36.6 (29–40.2)	0.0001	36.6 (29–44.1)	0.0003	22.3 (12.7–31.3)	0	13.55 (6.425–24.2)	0.0002	0.0007
Memory T-cell profile									
CD4^+^ Naïve T cells	41.5 (22.7–57.2)	ns	32.2 (22.7–56.4)	ns	NA		22.4 (3.95–47.3)	NA	
CD4T_CM_ cells	34.1 (23.1–41.5)	ns	38.6 (28.1–43.8)	0.0372			24.4 (8.65–40.1)		
CD4^++^ T_EMRA_ cells	1.5 (0.9–2.7)	0.0001	1.8 (0.9–2.6)	0.0001			26.6 (14.3–38.2)		
CD4^+^effector T cells	21.5 (11.1–30.2)	ns	21.3 (11.1–30.2)	ns			2.75 (0.7–40.8)		
CD8^+^ naïve T cells	37.4 (20.8–57.2)	0.0062	36.6 (21.3–47)	0.0108			15.1 (3.8–25.9)		
CD8^+^ T_CM_ cells	8.15 (5.2–13.8)	0.0029	8.6 (5.9–12.9)	0.0053			1.95 (0.9–7.95)		
CD8^+^ T_EMRA_ cells	20.7 (9.6–41.3)	ns	36.5 (12–44)	ns			17.3 (11.8–32.8)		
CD8^+^effector T cells	21.5 (12.3–32.7)	0.0005	21.4 (13.5–28.2)	0.0005			45.6 (26.5–64.8)		

ns, nonsignificant; NA, not available. Comparison of immune cell profiles among total Omicron COVID-19 patients group (*n* = 19), vaccinated Omicron-infected patients group (*n* = 15), total mild SARS-CoV-2 patients group (2020) (*n* = 45) and uninfected healthy controls (*n* = 18). Percentage of each cell type is represented as median (IQR), *p* value <0.05 is considered significant, *p* value ^a^: total Omicron COVID-19 patients vs. uninfected controls, *p* value ^b^: vaccinated Omicron COVID-19 patients group vs. uninfected controls, *p* value ^c^: total mild SARS-CoV-2 patients (2020) vs. uninfected controls, *p* value ^d^: total Omicron COVID-19 patients group vs. total mild SARS-CoV-2 patients group (2020), *p* value ^e^: vaccinated Omicron-infected patients group vs. total mild SARS-CoV-2 patients (2020) group.

**Table 3:** Spearman correlation analysis in Omicron COVID-19 patients.

Cell types	*R* value	*p* Value
CD4^+^ Naïve memory T cells	−0.4995	0.0294
CD8^+^Terminally differentiated memory T cells	0.5034	0.028
Memory B cells	0.6127	0.0053

Spearman correlation analysis among assessed parameters in patients with Omicron COVID-19 infection. Associations were tested for significance with Spearman's rank correlation. A *p* value <0.05 was considered significant.

**Table 4:** Levels of cytokines/chemokines in patients with Omicron infection and uninfected controls.

Analytes	Omicron-infected patients (*n* = 19)	Omicron-infected patients vs. uninfected controls	Uninfected controls (*n* = 36)
Pro-inflammatory cytokines			
IL-1*β*	0.1 (0–0.3)	ns	0.1 (0–1.53)
IL-5	0.8 (0–0.16)	0.035	0.91 (0.1–2.32)
IL-6	0.1 (0–0.21)	0.000	0.08 (0–1.17)
IL-7	0.73 (0.33–1.31)	0.000	0.21 (0.1–1.94)
IL-9	2.15 (0.23–2.51)	0.000	2.17 (0.69–2.37)
IL-15	1 (0–1.89)	0.000	0.18 (0.1–3.12)
IL-17	0.5 (0.45–0.97)	ns	0.58 (0–1.9)
TNF-*α*	1.09 (0.56–1.73)	0.000	1.28 (0.1–1.95)
Anti-inflammatory cytokines			
IL-1ra	1.66 (1.57–2.1)	ns	1.38 (0.1–3.32)
IL-4	0.1 (0–0.4)	ns	0.09 (0–0.71)
IL-10	0.15 (0.06–0.74)	0.012	0.29 (0–1.61)
IL-13	0.1 (0–0.6)	ns	0.1 (0–3.12)
TH1 cytokines			
IFN-*γ*	0.14 (0.05–0.55)	0.000	0.13 (0–1.9)
IL-2	0.08 (0–0.1)	0.000	0.71 (0–1.43)
IL-12(p70)	0.38 (0.31–1.24)	ns	0.27 (0–1.81)
Chemokines			
Eotaxin	0.84 (0–1.77)	0.000	1.50 (0–1.98)
CCL-2/ MCP-1(MCAF)	0.54 (0–1.3)	0.000	1.73 (0.05–2.2)
CCL-3/ MIP-1*α*	0.1 (0–0.5)	0.000	0.41 (0–1.28)
CCL-4/MIP-1*β*	1.7 (0.28–1.86)	0.000	2.07 (0.1–2.33)
CCL-5/RANTES	2.44 (0–3.16)	0.000	3.43 (0.86–4.02)
IL-8	0.05 (0–0.56)	0.000	1.25 (0–2.03)
CXCL-10/IP-10	1.95 (0.83–3.18)	0.010	1.8 (0.1–2.23)
Growth factors			
FGF basic	0.68 (0.6–0.97)	0.000	1.07 (0.1–2.4)
G-CSF	1.15 (0.83–2.12)	0.000	2 (1.39–2.61)
GM-CSF	0.1 (0–0.2)	0.000	0.11 (0–1.13)
VEGF	1.45 (0–1.55)	0.000	2.36 (1.83–2.68)
PDGF-bb	1.56 (0.51–2.5)	0.000	2.23 (1.03–2.97)

Values for cytokines and chemokines are presented as median (IQR). Log_10_ pg/mL (range); *p* < 0.05 considered significant, ns, nonsignificant.

**Table 5:** ROC characteristics of the cytokines analysis in Omicron COVID-19 patients.

Analytes	Cutoff (pg/mL)	Sensitivity (%)	Specificity (%)	AUC value	*p*-Value
IL-6	0.33	95	91.67	0.9056	<0.0001
IFN-*γ*	3.48	100	86.11	0.9583	<0.0001
CCL-2/ MCP-1(MCAF)	13.78	100	97.22	0.975	<0.0001
CCL-3/ MIP-1*α*	0.25	100	88.89	0.9569	<0.0001
CCL-4/MIP-1*β*	69.53	100	97.22	0.9722	<0.0001
CCL-5/RANTES	4,547.35	100	91.67	0.9722	<0.0001
IL-8	2.61	100	97.22	0.9722	<0.0001

Receiver operating characteristic (ROC) of the cytokines IL-6, IFN-*γ*, CCL-2/ MCP-1 (MCAF), CCL-3/ MIP-1*α*, CCL-4/MIP-1*β* CCL-5/RANTES, and IL-8 in plasma of Omicron-infected patients and uninfected controls. Levels of all analytes were analyzed post-log_10_ transformation of the observed concentrations of individual cytokine/chemokine/growth factors. The Mann–Whitney *U* test was used for numerical data for comparisons among the study groups. A *p*-value of <0.05 was considered significant. ROC analysis was performed using GraphPad Prism 8 software, AUC, area under the curve.

## Data Availability

The immunophenotyping, SARS-CoV-2-specific T-cell response, the cytokine data ROC curves, and analysis data in Omicron-infected patients and uninfected controls used to support the findings of this study are included within the article.

## Supplementary Materials

Supplementary 1 NGS results, S1 RBD, N protein, COVID KAWACH ELISA and PRNT levels in patients with Omicron infection (*n* = 19).

Supplementary 2 Schematic representation of the study design and characteristics of the study subjects, the NGS results, S1 RBD, N protein, COVID KAWACH ELISA, and PRNT levels in patients with Omicron infection used to support the findings of this study are included within the supplementary information files. Figure S1: schematic representation of the study design. Figure S2: schematic representation of characteristics of the study subjects.
