# Supplementary material for: Broadly Reactive SARS-CoV-2-Specific T-Cell Response and Participation of Memory B and T Cells in Patients with Omicron COVID-19 Infection
Source: J Immunol Res. 2023 Oct 17;2023:8846953. doi: 10.1155/2023/8846953 (PMC10597734; doi:10.1155/2023/8846953)
Supplement: Supplementary 1 — NGS results, S1 RBD, N protein, COVID KAWACH ELISA and PRNT levels in patients with Omicron infection (n = 19). [file 8846953.f1.docx]

**Broadly reactive SARS-CoV-2 specific T cell response and participation of memory B and T cells in patients with Omicron COVID-19 infection**

Pragya D. Yadav ^1^, Rima R. Sahay ^1$^, Sukeshani Salwe ^1$,^ Diptee Trimbake ^1$^, Prasad Babar^1^, Gajanan N. Sapkal^1^, Gururaj R. Deshpande ^1^, Kiran Bhise ^2^, Anita M. Shete ^1^, Priya Abraham^1^, Anuradha S. Tripathy ^1 *^

**Supplementary Table SI**

**Supplementary Table SI: NGS results, S1 RBD, N protein, COVID KAWACH ELISA &PRNT levels in patients with Omicron infection (n=19)**

| Patient Id | | NGS result | S1 RBD anti-IgG ELISA | N protein anti-IgG ELISA | COVID KAWACH anti-IgG ELISA | | PRNT titre | | | | |
| --- | --- | --- | --- | --- | --- | --- | --- | --- | --- | --- | --- |
|  |  |  | **Titre** | **Titre** | **Titre** | **B.1** | | **Omicron** | **Delta** | **Beta** | **Alpha** |
| 1 | B.1.1.529 | | >3200 | >3200 | 3200 | 4461 | | 2136 | 2649 | 3234 | 450 |
| 2 | B.1.1.529 | | <50 | <50 | <100 | 0.09 | | 14 | 0.09 | 13 | 1514 |
| 3 | B.1.1.529 | | >3200 | <50 | 400 | 5274 | | 6420 | 6541 | 7129 | 3580 |
| 4 | B.1.1.529 | | 2000 | 3200 | 3200 | 1337 | | 924 | 1500 | 780 | 534 |
| 5 | B.1.1.529 | | 1600 | 3200 | 3200 | 4035 | | 1923 | 1437 | 1204 | 450 |
| 6 | B.1.1.529 | | 2300 | >3200 | 3200 | 5832 | | 2500 | 2328 | 2873 | 903 |
| 7 | B.1.1.529 | | 3200 | 3200 | 3200 | 6894 | | 1825 | 5052 | 6587 | 895 |
| 8 | B.1.1.529 | | 1000 | 50 | 100 | 5832 | | 2500 | 4253 | 1522 | 1500 |
| 9 | Probable Omicron | | 2800 | 200 | 400 | 6030 | | 3206 | 3430 | 2358 | 1500 |
| 10 | B.1.1.529 | | 100 | <50 | 100 | 44 | | 178 | 0.09 | 131 | 190 |
| 11 | BA.1 | | 800 | 50 | 100 | 722 | | 593 | 728 | 693 | 378 |
| 12 | BA.1 | | 3000 | 50 | 100 | 5101 | | 1923 | 2046 | 2989 | 5052 |
| 13 | BA.1 | | 2000 | <50 | 6400 | 1559 | | 674 | 608 | 589 | 378 |
| 14 | Probable Omicron | | <50 | <50 | 100 | #NA | | | | | |
| 15 | Probable Omicron | | 3000 | 50 | <100 |  |  |  |  |  |  |
| 16 | Probable Omicron | | 3200 | 3200 | 1600 |  |  |  |  |  |  |
| 17 | Probable Omicron | | 2000 | 50 | <100 |  |  |  |  |  |  |
| 18 | Probable Omicron | | >3200 | <50 | 100 |  |  |  |  |  |  |
| 19 | Probable Omicron | | 2000 | <50 | 100 |  |  |  |  |  |  |

#NA: not available
