# Supplementary material for: Broadly Reactive SARS-CoV-2-Specific T-Cell Response and Participation of Memory B and T Cells in Patients with Omicron COVID-19 Infection
Source: J Immunol Res. 2023 Oct 17;2023:8846953. doi: 10.1155/2023/8846953 (PMC10597734; doi:10.1155/2023/8846953)
Supplement: Supplementary 2 — Schematic representation of the study design and characteristics of the study subjects, the NGS results, S1 RBD, N protein, COVID KAWACH ELISA, and PRNT levels in patients with Omicron infection used to support the findings of this study are included within the supplementary information files. Figure S1: schematic representation of the study design. Figure S2: schematic representation of characteristics of the study subjects. [file 8846953.f2.pptx]

## Slide 1
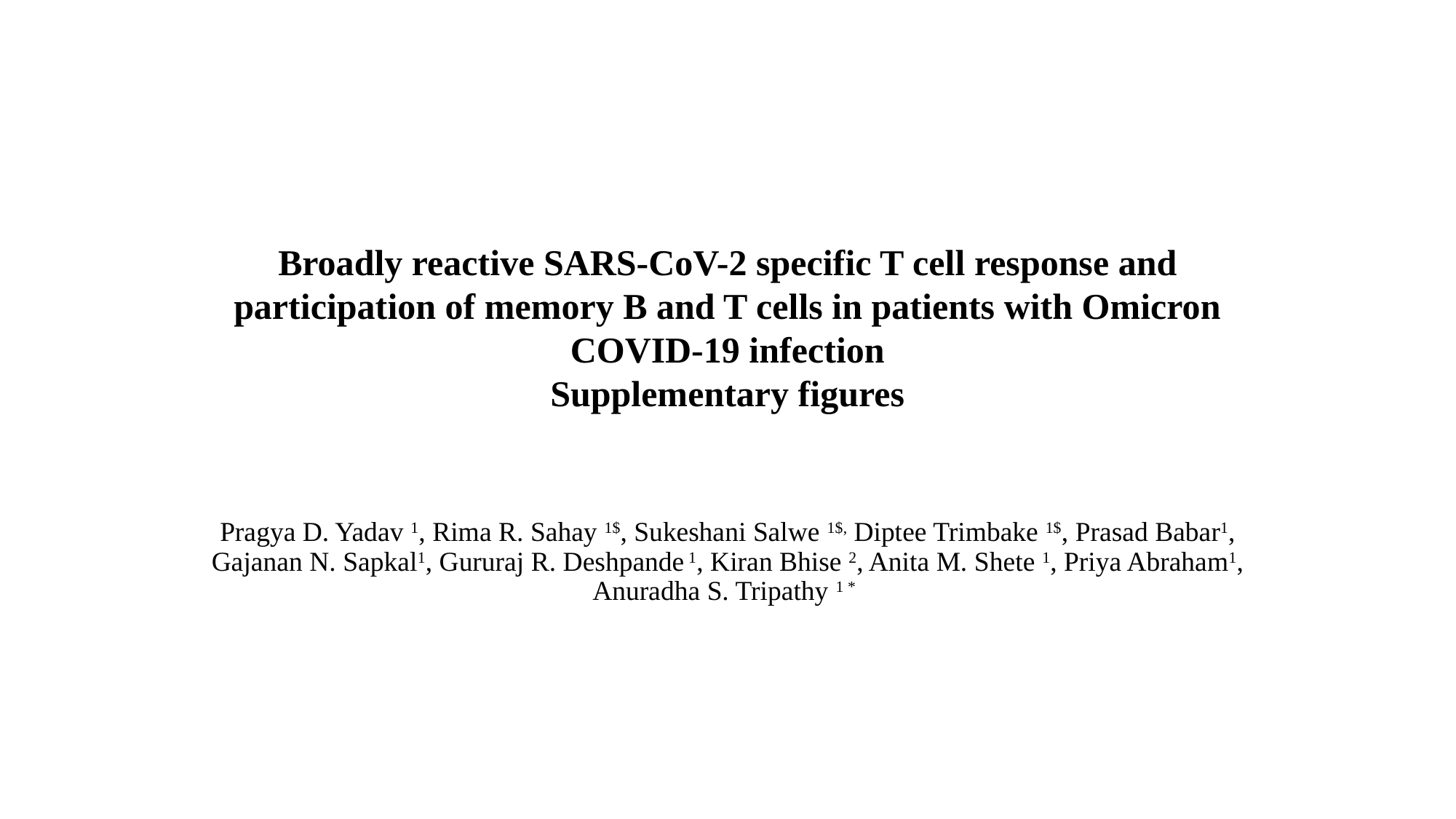

# Broadly reactive SARS-CoV-2 specific T cell response and participation of memory B and T cells in patients with Omicron COVID-19 infection Supplementary figures
Pragya D. Yadav 1, Rima R. Sahay 1$, Sukeshani Salwe 1$, Diptee Trimbake 1$, Prasad Babar1, Gajanan N. Sapkal1, Gururaj R. Deshpande 1, Kiran Bhise 2, Anita M. Shete 1, Priya Abraham1, Anuradha S. Tripathy 1 *

## Slide 2
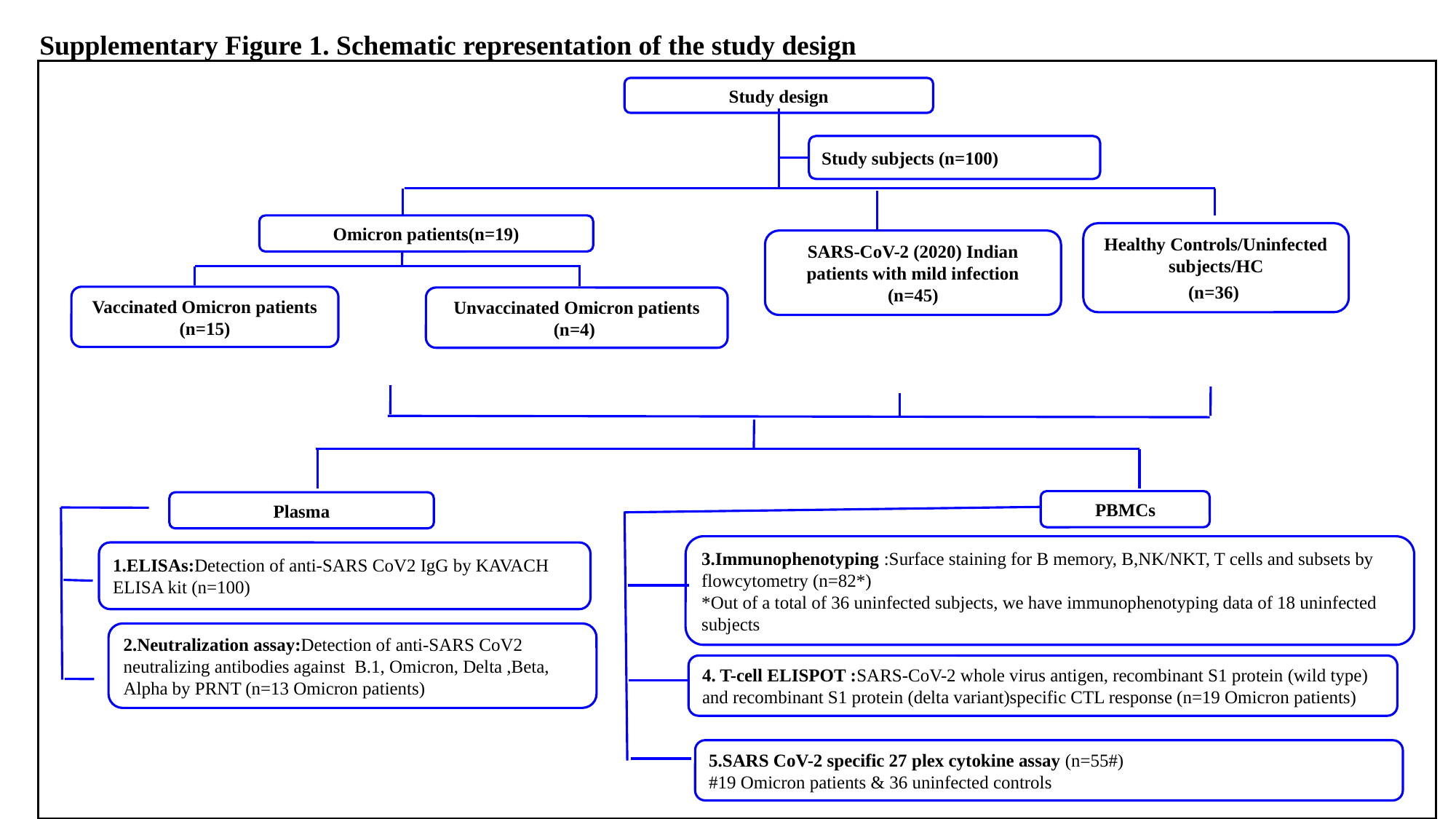

Supplementary Figure 1. Schematic representation of the study design
Study design
Omicron patients(n=19)
Healthy Controls/Uninfected subjects/HC
(n=36)
SARS-CoV-2 (2020) Indian patients with mild infection
(n=45)
Vaccinated Omicron patients (n=15)
Unvaccinated Omicron patients (n=4)
PBMCs
3.Immunophenotyping :Surface staining for B memory, B,NK/NKT, T cells and subsets by flowcytometry (n=82*)
*Out of a total of 36 uninfected subjects, we have immunophenotyping data of 18 uninfected subjects
4. T-cell ELISPOT :SARS-CoV-2 whole virus antigen, recombinant S1 protein (wild type) and recombinant S1 protein (delta variant)specific CTL response (n=19 Omicron patients)
5.SARS CoV-2 specific 27 plex cytokine assay (n=55#)
#19 Omicron patients & 36 uninfected controls
Plasma
1.ELISAs:Detection of anti-SARS CoV2 IgG by KAVACH ELISA kit (n=100)
2.Neutralization assay:Detection of anti-SARS CoV2 neutralizing antibodies against B.1, Omicron, Delta ,Beta, Alpha by PRNT (n=13 Omicron patients)
Study subjects (n=100)

## Slide 3
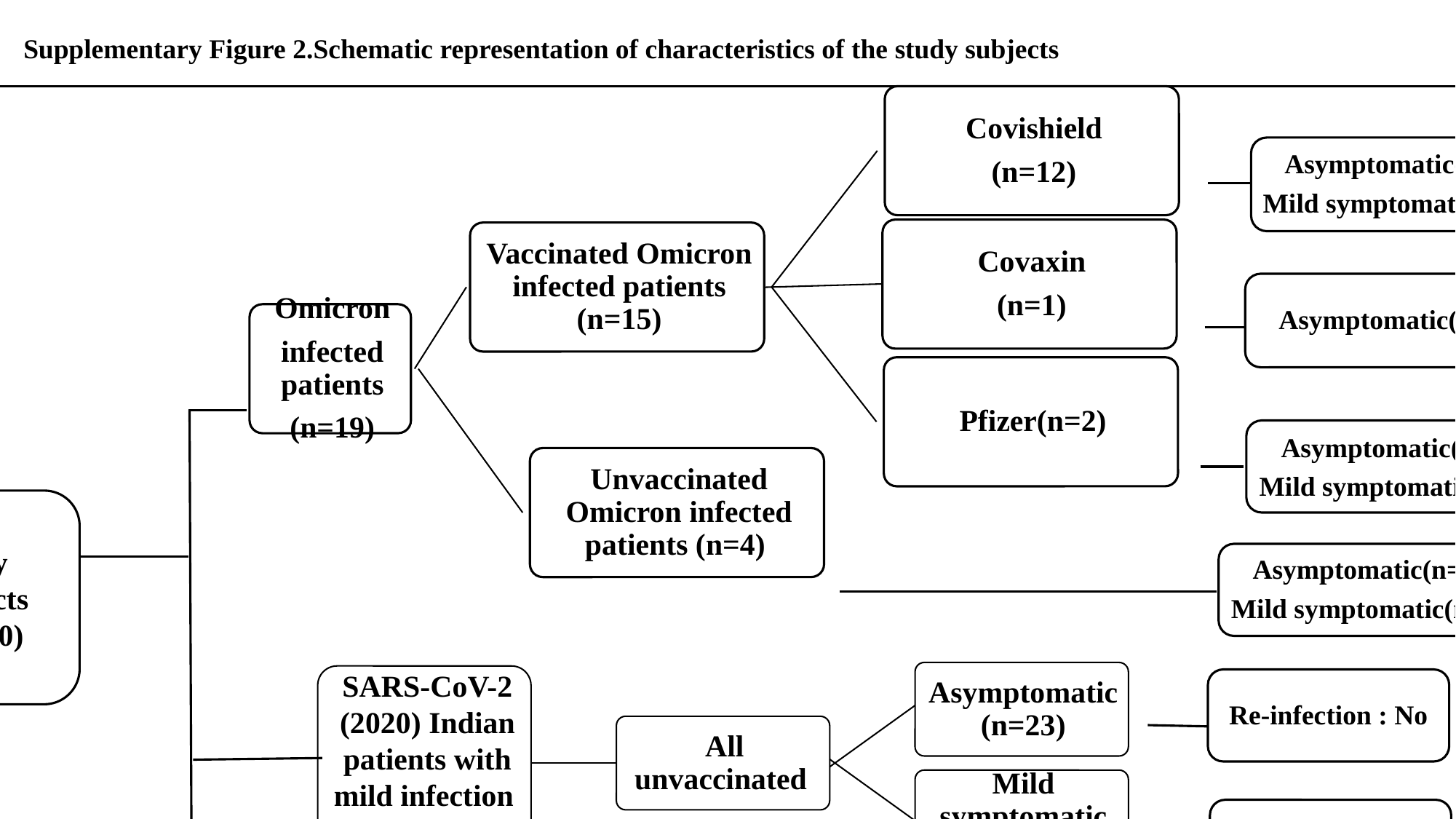

Supplementary Figure 2.Schematic representation of characteristics of the study subjects
Re-infection(n=2)
Asymptomatic(n=9)
Mild symptomatic(n=3)
Re-infection(n=1)
Asymptomatic(n=1)
Re-infection : No
Asymptomatic(n=1)
Mild symptomatic(n=1)
Asymptomatic(n=3)
Mild symptomatic(n=1)
Re-infection : No
Study
Subjects
(n=100)
Uninfected/
Healthy Controls
(n=36)
All unvaccinated
All anti-SARS-CoV-2 IgG antibody negative
Re-infection : No
Re-infection : No
